# From noticing to reflection: A qualitative exploration of rapid cycle deliberate practice effects on electrocardiographic monitoring judgment in critical cardiac care nurses

**DOI:** 10.1371/journal.pone.0353168

**Published:** 2026-07-06

**Authors:** Jun Xie, Wenyu Yue, Xiaoyan Wu, Lili Wu

**Affiliations:** 1 Nursing Department, Sir Run Run Shaw Hospital, Zhejiang University School of Medicine, Hangzhou, China; 2 School of Nursing, Zhejiang Chinese Medical University, Hangzhou, Zhejiang, China; University of Verona, ITALY

## Abstract

**Background:**

Electrocardiographic interpretation competency among critical cardiac care nurses remains inadequate despite technological advances, creating patient safety concerns and alarm fatigue. Traditional nursing education utilizing didactic knowledge transmission has proven insufficient for developing time-critical electrocardiographic interpretation skills. Rapid cycle deliberate practice emerges as an innovative methodology integrating deliberate practice principles, immediate feedback, and psychologically safe environments through pause-coach-resume mechanisms.

**Methods:**

Semi-structured interviews utilized Tanner’s clinical judgment model as the questioning framework, supplemented by field observations. Interviews explored experiences around the noticing-interpreting-responding-reflecting continuum, with analysis conducted using NVivo software following van Manen’s methodology.

**Results:**

Four interconnected themes emerged: shifting perceptual focus from fragmented data perception toward holistic pattern recognition; shifting cognitive models from mechanical matching toward integrating intuition and evidence; evolving response patterns from hesitant reactivity toward confident proactivity; and redefined professional role encompassing new professional identity, ethical responsibility, and continuous learning commitment.

**Conclusions:**

Analysis through Tanner’s clinical judgment framework suggested perceived multidimensional shifts within critical cardiac care nurses’ clinical judgment processes, as reflected in participants’ descriptions of changes transcending technical skill acquisition to encompass reconceptualizations of clinical reasoning, professional identity, and ethical responsibility. Notably, the psychologically safe environment created through the pause-coach-resume mechanism appeared central to enabling these perceived shifts. These findings indicate that effective competency development requires educational approaches addressing cognitive, behavioral, and identity dimensions simultaneously, with implications for institutional integration of rapid cycle deliberate practice into staff development and future longitudinal investigation of its effectiveness.

## 1 Introduction

Cardiovascular disease remains the leading cause of mortality worldwide, situating critical cardiac care units (CCUs) as high-acuity environments where nursing competencies are closely associated with patient survival outcomes and broader public health indicators [1]. Within these settings, accurate electrocardiographic interpretation serves as a cornerstone of therapeutic efficacy, as delayed recognition of cardiac rhythm abnormalities may precipitate irreversible hemodynamic compromise within minutes [2]. Contemporary nursing practice reveals a persistent paradox: while technological sophistication in cardiac monitoring continues to advance, nursing competency in interpreting complex and atypical electrocardiographic patterns remains inadequate, particularly when confronting the nuanced presentations characteristic of critical cardiac events [3]. This deficit is compounded by alarm fatigue, wherein the overwhelming frequency of false alarms may systematically erode nursing vigilance and diagnostic acumen [4]. Traditional educational approaches, rooted in didactic knowledge transmission and episodic skill rehearsal, have demonstrated limited capacity to address the contextual and time-critical demands of electrocardiographic interpretation in contemporary critical care settings [5].

The Chinese healthcare landscape presents contextual factors that compound challenges in critical care nursing education. Despite national policies establishing favorable nurse-to-patient ratios, the cognitive and emotional demands inherent in critical cardiac care tend to create workload intensities that exceed the supportive capacity of numerical staffing adequacy [6]. Elevated nursing turnover within intensive care environments perpetuates a cycle wherein experienced practitioners migrate to less demanding positions, leaving predominantly novice nurses to manage the most clinically complex patient populations [7]. While national healthcare policy reflects a commitment to advanced nursing education, the translation of policy directives into standardized, evidence-based training protocols remains inconsistently implemented across institutions [8]. Current educational frameworks establish broad competency expectations without providing specific methodological guidance for skill development, contributing to notable inter-institutional variation in training quality. Traditional curricula continue to prioritize theoretical knowledge transmission over practical competency development, contributing to persistent gaps between didactic learning and clinical performance [9].

Rapid Cycle Deliberate Practice (RCDP) represents an educational methodology that reconceptualizes skill acquisition through the integration of deliberate practice principles, mastery learning frameworks, immediate performance feedback, and psychologically safe learning environments [10]. Distinguished from conventional training approaches relying upon post-hoc reflection and delayed corrective guidance, RCDP employs an iterative “pause-coach-resume” mechanism enabling real-time intervention during skill demonstration and facilitating immediate error correction within structured educational contexts [11]. This approach is particularly relevant to electrocardiographic interpretation, which constitutes a hybrid cognitive-procedural skill characterized by pattern recognition demands, contextual decision-making requirements, and intuitive response necessities that resist traditional theoretical instruction [12]. The complexity of electrocardiographic monitoring extends beyond technical pattern identification to encompass clinical correlation, hemodynamic assessment, and therapeutic prioritization, competencies that develop through experiential learning rather than abstract knowledge acquisition [13]. These attributes position RCDP as a structurally distinct alternative to the predominantly didactic training approaches prevalent in Chinese CCU nursing education, where the iterative, feedback-intensive mechanisms necessary for contextual skill development remain inconsistently available [8,9].

Tanner’s Clinical Judgment Model [14] provides a theoretical framework for understanding the cognitive processes underlying nursing clinical decision-making through four interconnected phases: noticing, interpreting, responding, and reflecting [15]. The core mechanisms of RCDP align structurally with each of these phases: the “pause” targets noticing by redirecting learner attention toward clinically salient features at moments of perceptual error; “coaching” scaffolds interpreting through guided analytical exchange that supports more accurate pattern recognition; and the immediate “resume” enables corrected responding within the same practice episode. These mechanisms collectively compress the reflecting phase into real-time learning embedded within the practice encounter itself, rather than deferred to retrospective debriefing. This structural correspondence suggests that integrating RCDP with Tanner’s framework may offer a theoretically coherent basis for accelerating clinical judgment development in electrocardiographic interpretation education.

Current literature reveals substantial limitations in understanding the mechanisms underlying nursing competency transformation, with predominant attention directed toward intervention outcomes rather than learning processes. Research examining the integration of clinical judgment frameworks with innovative pedagogical approaches represents a critical gap in nursing education theory, particularly within Chinese healthcare contexts. This investigation addresses these gaps through two primary objectives: (1) to explore the lived experiences of CCU nurses participating in RCDP training structured according to Tanner’s Clinical Judgment Model, and (2) to illuminate the subjective processes associated with electrocardiographic interpretation competency development. The phenomenological approach, specifically van Manen’s interpretive phenomenology, provides methodological alignment for exploring the subjective learning experiences that characterize the transition from novice rule-application toward expert clinical judgment. The anticipated contributions include advancing theoretical understanding of clinical judgment applications in specialized nursing contexts, informing evidence-based training framework development, and providing empirical foundations for nursing education policy advancement.

## 2 Methodology

### 2.1 Aim

This investigation aimed to elucidate the lived experiences and clinical judgment transformation processes of critical care unit nurses participating in rapid cycle deliberate practice training grounded in Tanner’s clinical judgment model through phenomenological inquiry.

### 2.2 Study design

#### 2.2.1 Phenomenological design and theoretical framework.

The study employed a qualitative phenomenological design informed by van Manen’s interpretive phenomenological framework [16], which emphasizes hermeneutic understanding and articulation of essential meanings within “lifeworld” experiences. Tanner’s clinical judgment model [14] structured the interview guide around the “noticing-interpreting-responding-reflecting” continuum; its application to thematic organization occurred after inductive coding reached stabilization. The primary investigator, a female clinical educator with qualitative research training and NVivo proficiency, conducted all interviews. The semi-structured interview guide underwent review by two qualitative research experts and pilot testing to ensure question clarity and logical sequencing, with wording adjustments based on pilot feedback, adhering to established standards for reporting qualitative research [17].

#### 2.2.2 Reflexivity and positionality.

The primary investigator’s concurrent role as RCDP implementer and interviewer necessitated attention to positionality and reflexivity throughout the study. Her programmatic investment as a clinical educator generated an a priori assumption of positive RCDP outcomes, carrying potential to shape both data collection and interpretive judgment. To bracket this preconception [18], a reflexive journal was maintained throughout data collection and analysis, documenting interpretive inclinations, emotional responses, and the reasoning underlying analytical decisions; entries were periodically reviewed to identify assumptions warranting suspension prior to subsequent interviews and coding cycles.

At the interview level, all questions employed open-ended, non-directive phrasing to minimize confirmatory framing and socially desirable responding. Prior to each interview, the investigator clarified her researcher identity and study objectives, distinguishing her investigative role from her implementation role and emphasizing voluntary participation unrelated to performance evaluation. Analytically, peer debriefing with co-authors provided critical examination of emerging thematic interpretations and challenge to potentially biased conclusions. This combination of reflexive documentation, procedural transparency, and collaborative scrutiny constitutes the primary mechanisms through which the trustworthiness of the data and interpretations was pursued [19].

#### 2.2.3 RCDP intervention protocol.

Referencing the RCDP framework described in Tang et al. [20], a three-phase, station-based RCDP training program targeting ECG monitoring competency in CCU nursing was designed and implemented. In Phase 1, informed by expert consensus, clinical guidelines, evidence summaries, and institutional competency standards, ECG interpretation and emergency response competencies were structured into four training stations: rhythm identification and rate calculation; waveform morphology analysis of P wave configuration, QRS complex characteristics, and ST-T segment evaluation; arrhythmia classification and clinical significance assessment; and emergency response decision-making with interdisciplinary communication. In Phase 2, station-specific performance objectives were established per institutional competency criteria, with nurses engaging in repeated deliberate practice under assessor observation; immediate corrective feedback was delivered via the pause-coach-resume mechanism until each objective was achieved. In Phase 3, clinical case scenarios were embedded at each station; following foundational proficiency, complexity was progressively escalated through atypical ECG presentations and multi-task clinical scenarios, with time constraints and accuracy thresholds introduced to facilitate transition toward advanced clinical judgment.

### 2.3 Sample and setting

The study was conducted in the cardiac critical care unit of a tertiary general hospital in Zhejiang Province, China. Participants comprised registered CCU nurses who completed RCDP training, recruited through convenience sampling, which facilitated efficient access to participants with direct RCDP experience although it carries an inherent risk of selection bias given the single-site context [21]. Inclusion criteria were: (1) direct participation in cardiac monitoring and bedside care within the study CCU; (2) completion of RCDP training during the research period; (3) minimum three years of clinical experience including at least one year of continuous CCU service; (4) clear Mandarin expression with consent for audio recording. Exclusion criteria included: (1) inability to complete interviews or follow-up sessions due to rotation, sick leave, or transfer; (2) unwillingness to participate in clinical observation or audio recording.

**Fig 1:**
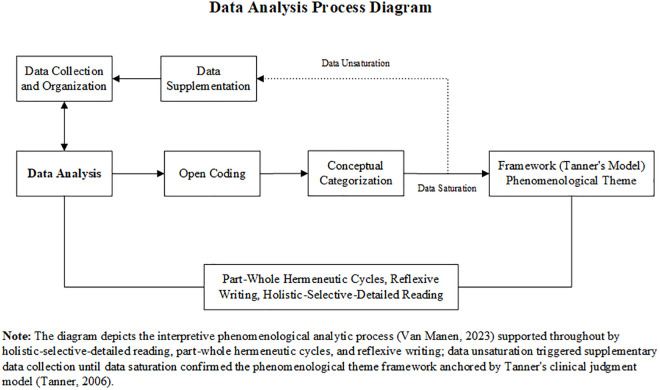
Data analysis process diagram.

Sample size followed data saturation principles, declared when consecutive interviews yielded no new codes. Two researchers independently coded each transcript and compared resulting meaning units against the cumulative coding framework; saturation was declared when three consecutive interviews yielded no new meaning units or thematic dimensions relevant to the research aim, documented through analytical memos and a saturation tracking log reviewed at each coding cycle (Fig 1). Interviews were scheduled during off-duty or post-shift periods in quiet locations, coordinated with the nurse manager to minimize departmental interference and protect participant privacy. Participants were recruited via posted materials and nurse manager referrals, with informed consent and scheduling conducted in the unit’s private study room or conference room, excluding third-party observers [22]. The sample achieved maximum variation across experience levels, professional ranks, and educational backgrounds to enhance experiential diversity [23]; no withdrawals occurred.

### 2.4 Data collection

Research implementation occurred between April and July 2025, during which RCDP training (Detailed in 2.2.3) was organized and completed alongside semi-structured individual interviews and field observations. The semi-structured interview guide was constructed with reference to Tanner’s model [14] to cover the “noticing-interpreting-responding-reflecting” continuum across interview topics, with the interview guide detailed in Supplement File 1. Interviews were conducted in quiet, private conference rooms with full audio recording following consent and subsequent verbatim transcription. To enrich contextual understanding and behavioral cues, researchers implemented accompanying clinical field observations within the CCU while conducting brief, unstructured situational exchanges within the natural contexts of training and clinical interactions, simultaneously generating field notes and reflexive memos to document observational context and support analytical reflexivity.

The study included thirteen CCU nurses, with individual interviews averaging approximately 43 minutes. Researchers documented contextual cues, non-verbal behaviors, and investigator reflections through immediate and delayed field notes completed on the day of and the day following each interview respectively. A minority of participants received supplementary 15–20 minute interviews within one week to verify key narratives or complete contextual details. Thematic summaries rather than verbatim transcripts were returned to participants for credibility feedback following preliminary theme development, an approach adopted for logistical feasibility that precluded transcript-level verification of interpretive accuracy. Audio recordings served research purposes exclusively and entered restricted-access encrypted storage following transcription and verification completion.

### 2.5 Data analysis

Analysis commenced immediately upon completion of the first interview, utilizing NVivo 12.0 software following Edwards-Jones’s methodological guidelines [24], with two researchers independently conducting initial open coding within NVivo, comparing discrepancies and achieving consensus through discussion, supplemented by third-party review from a qualitative methods specialist when necessary. Methodologically, analysis adhered to van Manen’s interpretive phenomenological research activities [16], pursuing “essential meanings” within experiential texts through holistic, selective, and detailed reading approaches while completing meaning generation through iterative “part-whole” hermeneutic cycles (see 2.3, Fig 1). Field notes were consulted to contextualize interpretive decisions and were excluded from the NVivo coding structure. Initial coding was conducted inductively without reference to Tanner’s model, allowing meaning units and conceptual categories to emerge directly from participant narratives. Tanner’s clinical judgment model [14] was introduced only after theme stabilization to provide conceptual anchoring for organizing themes and subthemes within the “noticing-interpreting-responding-reflecting” sequence. To ensure that emergent meanings were not constrained by the framework, the research team compared the inductively derived thematic structure against the Tanner-organized version, retaining data-driven labels and boundaries where discrepancies arose, with decisions documented in coding memos. Continuous reflexive writing, research journaling, and peer discussion accompanied the analytic process.

The coding tree presented hierarchical nodes, progressively aggregating from foundational meaning units to conceptual categories, ascending to phenomenological themes and subthemes (illustrated in Table 1 using Participant 1 as a representative exemplar). Following theme stabilization, member checking sessions utilized summarized thematic frameworks and representative quotations to solicit participant feedback, with adjustments to theme labels and boundaries made accordingly. Complete audit trails including coding memos, decision logs, and versioned node structures remained preserved in restricted repositories.

**Table 1:** Coding progression example.

Interview transcript excerpt	Meaning units	Conceptual category	Theme/subtheme
“Before, when I heard an alarm, my first reaction was to see which patient’s bed it was and then rush over to look at the numbers on the monitor. I basically just looked at the most fundamental indicators like heart rate, blood pressure, and oxygen saturation to see which one was out of range.” (Q1, P1)	¹Prioritizing isolated numerical parameters; ²Reacting to alarm sounds rather than clinical patterns; ³Sequential checklist approach to ECG review	Alarm-reactive numerical fixation	Theme 1: Shifting Perceptual Focus/Fragmented Data Perception
“Now, when identifying an ECG abnormality, the first thing I ‘latch onto’ is no longer a single waveform, but the overall ‘rhythm’ and ‘waveform characteristics’ of the entire ECG.” (Q5, P1)	⁴Shifting from isolated waveforms to overall rhythm; ⁵Integrating multiple ECG elements simultaneously; ⁶Perceiving holistic waveform characteristics	Integrated pattern recognition	Theme 1: Shifting Perceptual Focus/Holistic Pattern Perception
“When my intuitive judgment doesn’t perfectly match the standard procedure, I definitely struggle with it internally. After all, I used to strictly follow the rules, and suddenly having to trust my gut is a bit nerve-wracking. But I try to trust my feeling first, and then quickly verify it.” (Q6, P1)	⁷Internal tension between intuition and protocol; ⁸Developing trust in experiential judgment; ⁹Sequential verification of intuitive assessments	Intuition-evidence integration	Theme 2: Shifting Cognitive Models/Integrating Intuition and Evidence

### 2.6 Ethical considerations

This study received ethical approval from the Ethics Committee of Sir Run Run Shaw Hospital, Zhejiang University School of Medicine (Approval number: 2025-2306-01, Clinical trial number: not applicable.) in accordance with the Declaration of Helsinki [22]. Written informed consent was obtained from all participants prior to data collection, with voluntary participation and the right to withdraw clearly communicated. All data were de-identified; electronic files were stored on an encrypted, password-protected device with restricted access, and paper materials were secured in locked cabinets.

## 3 Results

### 3.1 Participant characteristics and phenomenological overview

The initial stage (Interviews 1–7) generated meaning units spanning all four themes and the majority of subthemes. The intermediate stage (Interviews 8–10) yielded the remaining subthemes alongside dimensional variations that refined existing categories. The final stage (Interviews 11–13) produced no new meaning units or thematic dimensions across three consecutive interviews, and saturation was declared following collaborative team review.Table 2 provides a detailed summary of the saturation evidence across the three interview stages.

**Table 2:** Data Saturation Evidence Across Interview Stages.

Interview Stage	Thematic Development	Saturation Indicators	Analytical Memo Evidence
Early interviews (Interviews 1–7)	All four themes identified; majority of subthemes emerged	New meaning units generated across all interview domains; core thematic architecture established	Coding produced diverse meaning units across perceptual, cognitive, behavioral, and professional identity dimensions. Memos identified conceptual gaps in certain subthemes, necessitating continued sampling.
Later interviews (Interviews 8–10)	Remaining subthemes emerged; existing categories refined	Progressively fewer new meaning units; new contributions primarily represented dimensional variations within established themes	Meaning units enriched established categories with experiential nuances. Memos noted diminishing novelty and increasing confirmatory patterns across interviews.
Final three consecutive interviews (Interviews 11–13)	No new themes or subthemes identified	Three consecutive interviews yielded no new meaning units or thematic dimensions; framework confirmed as stable	Interviews produced experiential descriptions subsumed within the established thematic framework. Research team declared data saturation following peer review of the final coding structure.

**Fig 2:**
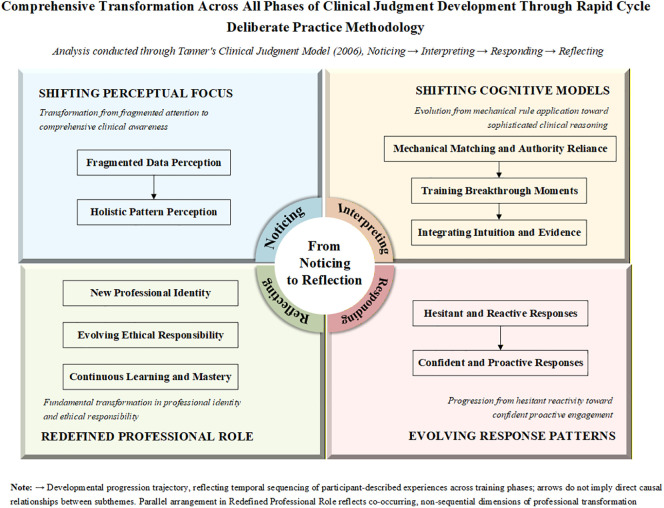
Phenomenological Analysis of RCDP Training Effects on Critical Cardiac Care Nurses Through Tanner’s Clinical Judgment Model.

Four interconnected themes represented a perceived comprehensive transformation in CCU nurses’ clinical judgment following RCDP training, organized per Tanner’s clinical judgment model: shifting perceptual focus from fragmented data perception toward holistic pattern recognition (noticing); shifting cognitive models from mechanical pattern matching toward integrating intuition and evidence (interpreting); evolving response patterns from hesitant reactivity toward confident proactivity (responding); and redefined professional role encompassing professional identity, ethical responsibility, and continuous learning commitment (reflecting). Fig 2 presents the thematic structure.

Thirteen CCU nurses participated, with ages ranging from 21 to 45 years, educational attainment from associate to master’s degrees, and clinical experience spanning 3–20 years. Professional titles included registered nurses, nurse practitioners, senior nurses, and associate chief nurses, across positions from staff nurses to deputy head nurses (Table 3). All quotations use participant (Px) and question (Qx) identification codes to maintain confidentiality.

**Table 3:** Participant demographic characteristics.

Number	Age Range	Education Level	Work Experience (Years)	Professional Title	Job Position
P1	31-35	Bachelor’s degree	9	Nurse Practitioner	Charge nurse
P2	21-25	Associate degree	3	Nurse	Staff nurse
P3	26-30	Bachelor’s degree	3	Nurse	Staff nurse
P4	31-35	Bachelor’s degree	8	Nurse Practitioner	Nurse educator
P5	21-25	Associate degree	3	Nurse	Staff nurse
P6	31-35	Bachelor’s degree	9	Senior Nurse	CCU specialist nurse
P7	26-30	Master’s degree	3	Nurse	Staff nurse
P8	31-35	Bachelor’s degree	8	Nurse Practitioner	CCU specialist nurse
P9	41-45	Bachelor’s degree	20	Associate Chief Nurse	Head nurse
P10	31-35	Master’s degree	8	Nurse Practitioner	Charge nurse
P11	26-30	Associate degree	6	Nurse Practitioner	Charge nurse
P12	26-30	Bachelor’s degree	3	Nurse	Staff nurse
P13	41-45	Associate degree	18	Senior Nurse	Deputy head nurse

**Note:** Age data are reported as range intervals rather than exact values to protect participant confidentiality.

### 3.2 Shifting perceptual focus

The reported transformation in nurses’ perceptual orientation represented, as described by participants, a fundamental reconceptualization of electrocardiographic monitoring from isolated data fragments to integrated pattern recognition.

#### 3.2.1 Fragmented data perception.

Prior to RCDP implementation, participants demonstrated predominantly reactive and fragmented approaches to electrocardiographic monitoring that prioritized isolated numerical parameters over comprehensive clinical assessment. Participants described mechanistically focused attention on discrete monitoring elements: *“Before, when I heard an alarm, my first reaction was to see which patient’s bed it was and then rush over to look at the numbers on the monitor”* (P1, Charge Nurse).

#### 3.2.2 Holistic pattern perception.

Following RCDP training, participants described a perceived transformation toward holistic pattern recognition that integrated multiple electrocardiographic elements into comprehensive clinical assessments, emphasizing overall rhythm characteristics and morphological patterns rather than isolated parametric values. Participants reported enhanced pattern recognition capabilities: *“Now, when identifying an ECG abnormality, the first thing I ‘latch onto’ is no longer a single waveform, but the overall ‘rhythm’ and ‘waveform characteristics’ of the entire ECG”* (P1, Charge Nurse).

Taken together, the perceptual shifts described across this theme carry meaningful implications for clinical attentiveness. Participants’ accounts suggest a reorientation of clinical gaze from the monitor as a terminal data source toward the monitor as a window onto the patient’s physiological state, reflecting a movement toward patient-centered interpretation in which rhythm recognition becomes inseparable from broader clinical awareness.

### 3.3 Shifting cognitive models

The reported evolution in cognitive processing models reflected, as described by participants, a perceived transformation from rigid rule-based thinking toward flexible clinical reasoning that integrated theoretical knowledge with experiential wisdom.

#### 3.3.1 Mechanical matching and authority reliance.

Participants initially employed cognitive approaches characterized by rigid textbook pattern adherence and excessive dependence on senior colleagues for clinical decision-making, creating persistent uncertainty when confronting atypical presentations. Nurses described inflexible cognitive processes as pattern-matching exercises failing to accommodate clinical complexity: *“My way of looking at an ECG was very mechanical, just matching it to textbook knowledge”* (P3, Staff Nurse).

#### 3.3.2 Training breakthrough moments.

During RCDP implementation, participants experienced distinct cognitive breakthrough moments facilitating transitions from mechanical rule application toward integrated clinical reasoning. These transformative experiences involved sudden recognition of previously overlooked clinical details: *“When I shifted my attention from individual waves to the overall rhythm...I suddenly noticed a detail I had never seen before...At that moment, I felt the whole ECG ‘came alive’ before my eyes”* (P1, Charge Nurse). These moments represented critical junctures at which participants began developing authentic clinical insight beyond superficial pattern recognition.

#### 3.3.3 Integrating intuition and evidence.

Post-training, participants described cognitive models demonstrating sophisticated integration of intuitive pattern recognition with systematic evidence validation. Participants described balanced cognitive frameworks combining immediate impressions with systematic confirmation: *“Now, when identifying an ECG abnormality, the first thing I ‘latch onto’ is an ‘intuitive judgment’ backed by ‘detailed confirmation’”* (P8, CCU Specialist Nurse).

Considered across sub-themes, the cognitive evolution participants described reflects a qualitative transformation in clinical reasoning style that extends beyond technical competency. The movement from textbook matching toward intuition-anchored, evidence-confirmed judgment suggests a reported capacity to navigate clinical ambiguity with greater flexibility, experienced by participants not merely as skill acquisition but as a reconceptualization of what it means to interpret an electrocardiogram in a critical care context.

### 3.4 Evolving response patterns

The reported transformation in response patterns reflected, as described by participants, a progression from hesitant, reactive behaviors toward confident, proactive clinical engagement, suggesting enhanced professional agency and evolving perceptions of professional identity.

#### 3.4.1 Hesitant and reactive responses.

Prior to RCDP training, participants demonstrated response patterns characterized by uncertainty, anxiety, and reactive behaviors reflecting insufficient clinical confidence and limited autonomous decision-making capabilities. Nurses experienced significant cognitive paralysis during complex scenarios: *“When things got complicated, like several alarms going off at once, honestly, my mind would just go blank”* (P1, Charge Nurse). This reactive pattern perpetuated professional insecurity cycles that participants described as hindering competency development.

#### 3.4.2 Confident and proactive responses.

Following RCDP implementation, participants described transformed response patterns characterized by enhanced confidence, proactive clinical engagement, and clearer communication of clinical findings. Participants reported increased confidence and professional authority: *“Now, I’ll say, ‘Patient in [Bed] 4’s monitor shows a coved ST-segment elevation...I recommend a doctor assess for acute myocardial infarction immediately’”* (P4, Nurse Educator).

Across the two response sub-themes, the transformation participants described reflects changes in professional comportment that extend beyond technical execution. Their accounts suggest that enhanced confidence was experienced as an expanded sense of professional agency, enabling more assertive clinical communication and proactive engagement, with implications for participants’ described positioning within interdisciplinary care teams.

### 3.5 Redefined professional role

The reported evolution in professional role conceptualization represented, in participants’ accounts, a perceived transformation from task-oriented execution toward autonomous clinical practice emphasizing professional accountability, ethical responsibility, and continuous learning commitment.

#### 3.5.1 New professional identity.

Participants experienced substantial transformation in professional identity from passive order followers toward active clinical collaborators contributing meaningfully to patient care decisions. This evolution was characterized by enhanced professional confidence and an expanded sense of nursing’s contribution to patient safety: *“I used to see myself as just an executor, doing whatever the doctor said. Now, I feel more like a ‘sentinel’ and a ‘collaborator’”* (P1, Charge Nurse). This identity transformation was described as facilitating enhanced job satisfaction and professional engagement within challenging clinical environments.

#### 3.5.2 Evolving ethical responsibility.

Participants described how enhanced clinical competency facilitated their expanded understanding of professional ethical responsibilities extending toward comprehensive patient advocacy and safety assurance. This described evolution encompassed increased willingness to challenge clinical decisions and accept responsibility for patient outcomes: *“This new ability has changed my understanding of nursing ethics; I believe nurses are not just there to follow orders, but to think proactively and be responsible for the patient’s safety”* (P1, Charge Nurse).

#### 3.5.3 Continuous learning and mastery.

Participants described sustained commitment to professional development and continuous learning reflecting intrinsic motivation for excellence. This orientation was exemplified by aspirations toward advanced knowledge acquisition: *“I feel like I’m in a rapid growth phase...To further improve my skills, I hope to delve deeper into electrophysiology to understand the pathological mechanisms behind the waveforms”* (P1, Charge Nurse).

Across these three sub-themes, the professional identity transformation participants described reflects a fundamental reconceptualization of nursing’s epistemic role within critical care settings. Their accounts indicate that enhanced competency was experienced as ethically generative, expanding participants’ sense of accountability beyond task completion toward active patient advocacy, with implications for understanding how clinical training may shape not only technical skill but also the normative commitments that characterize professional practice.

## 4 Discussion

This phenomenological investigation explored the lived experiences of critical cardiac care nurses following their participation in rapid cycle deliberate practice training, with analysis conducted through the theoretical lens of Tanner’s clinical judgment model [14]. The participants’ descriptions of their experiences suggest a multidimensional shift in nursing consciousness that appears in their accounts to extend beyond technical skill acquisition, encompassing what participants described as reconceptualizations of clinical reasoning processes.

The participants described a progression from fragmented data perception toward holistic pattern recognition that may reflect a notable shift in the perceptual dimensions of clinical judgment as experienced through RCDP. This perceived transformation aligns with Tanner’s conceptualization of enhanced “noticing” capabilities [14], wherein clinical nurses develop increasingly refined attentional mechanisms through combining clinical knowledge with experience, enabling them to rapidly identify clinically significant patterns and form responsive approaches [25]. The nurses’ accounts suggest that RCDP may have created conditions in their experience that promoted gestalt-based recognition processes, wherein they describe transitioning from sequential parameter evaluation toward simultaneous integration of multiple electrocardiographic elements. This perceptual evolution as described by participants may reflect the capacity to recognize meaningful clinical configurations rather than isolated data fragments [26].

The participants’ narratives describe a cognitive transformation from mechanical pattern matching toward integrated clinical reasoning that suggests, in their accounts, a perceptual reorganization in how they described accessing and applying clinical knowledge. Within Tanner’s framework [14], this evolution as articulated by the nurses appears to represent enhanced “interpreting” capabilities among intensive care nurses, whose ability to explain clinical condition changes improves with years of experience as they integrate theoretical knowledge with experiential wisdom [27]. The participants’ accounts indicate that RCDP may have facilitated cognitive flexibility in synthesizing multiple information sources while maintaining systematic verification [28], suggesting a perceived balance between intuitive recognition and systematic analytical verification. In the nurses’ descriptions of their experiences during rapid-cycle deliberate practice training, particularly through the distinctive “interrupt-and-restart” process when errors occur, they articulated what they perceived as critical cognitive transitions wherein they described beginning to perceive electrocardiographic presentations as integrated clinical narratives rather than disconnected technical data.

The participants described a transformation in response patterns from hesitant reactivity toward confident proactivity that in their accounts suggests enhanced self-efficacy perceptions appearing to influence both behavioral competency and professional identity. This evolution as experienced by the nurses aligns with Tanner’s “responding” phase [14], which encompasses not merely technical execution but emphasizes how nurses in their daily practice integrate clinical judgment with contextual adaptation [29]. The participants’ narratives suggest that RCDP may have created in their experience psychological safety conditions that enable risk-taking behaviors essential for competency development while simultaneously providing corrective feedback that builds confidence through successful performance experiences [30].

The participants’ descriptions of their redefinition of professional role suggest perhaps a meaningful perceived transformation articulated in their accounts, pointing toward shifts in professional identity that participants described as extending beyond individual skill development. The nurses’ narratives of enhanced ethical responsibility appear consistent with Tanner’s “reflecting” phase [14], which emphasizes the experiential learning processes that transform discrete clinical encounters into cumulative professional wisdom. The participants’ accounts suggest that their perceived clinical competency development appeared to be associated with a heightened sense of moral agency in complex care environments, with participants describing an increased orientation toward navigating hierarchical constraints while advocating effectively for patient welfare [31]. This professional identity shift as described by the nurses appeared in their accounts to reflect an evolving clinical confidence that participants perceived as integral to their professional self-concept.

The professional identity dimensions articulated in the participants’ accounts are consistent with scholarship proposing that skill acquisition and identity formation represent deeply intertwined processes in nursing education [32]. As nurses developed technical competence within authentic practice contexts, their accounts suggest a concurrent evolution in how they perceived their professional obligations and ethical responsibilities. The participants’ descriptions of heightened awareness regarding patient advocacy and moral responsibility in complex clinical situations resonate with the understanding of ethical sensitivity as a foundational component of ethical action, one that must be cultivated through education and accumulated clinical experience [33]. Interpreted through Tanner’s “reflecting” phase [14], these perceived shifts in ethical orientation may represent an emergent dimension of professional consciousness rather than a discrete educational outcome, suggesting that RCDP may have created conditions within which participants’ sense of ethical accountability developed alongside their technical proficiency.

The participants’ expressions of commitment to continuous learning revealed in their narratives suggest that RCDP may have been associated in their experience with intrinsic motivation mechanisms that sustain professional development beyond immediate educational objectives. This finding from the nurses’ accounts resonates with the established understanding that competency experiences tend to enhance intrinsic motivation for continued learning and mastery [34]. The participants’ articulated desire for advanced knowledge and academic engagement may indicate that their experience of effective clinical education appeared to foster an orientation toward self-directed professional development, wherein participants described assuming greater responsibility for their ongoing professional growth.

When analyzed through Tanner’s clinical judgment model [14], the participants’ descriptions suggest perceived enhancement across all phases of the framework, indicating that RCDP methodology may have addressed the interconnected nature of clinical reasoning processes rather than isolated skill components. The nurses’ accounts of the “pause-coach-resume” mechanism suggest it created conditions for immediate reflection and skill refinement within authentic practice contexts, facilitating what participants described as the integration of theoretical knowledge with practical application that may characterize expert clinical judgment development. These phenomenological findings collectively suggest that effective clinical education benefits from integrated approaches that simultaneously address cognitive, behavioral, and affective dimensions of professional competency, and that the deliberate practice principles embedded within RCDP, when combined with psychologically supportive learning environments, may enable practitioners to engage in the cognitive risk-taking that participants described as central to their perceived professional growth.

### 4.1 Practical implications

#### 4.1.1 Implications for clinical nursing education.

Participants’ accounts indicated a shift from alarm-reactive, parameter-focused monitoring toward integrated pattern recognition encompassing overall rhythm characteristics and morphological context, suggesting that training environments prioritizing isolated skill acquisition may be structurally misaligned with the perceptual orientation associated with competent clinical interpretation. Critical care units should therefore integrate RCDP training into routine staff development through simulation sessions during shift changes or downtime, using portable ECG simulators at nursing stations where experienced nurses provide immediate correction when interpretive errors occur and then resume the scenario. To cultivate this integrative perceptual capacity, units can create dedicated “ECG rounds” where nurses present challenging rhythms encountered during their shifts, with immediate group discussion that builds interpretive frameworks grounded in actual clinical exposure rather than decontextualized drill [13]. Monthly competency assessments should replace annual testing, requiring nurses to demonstrate rapid pattern recognition across common arrhythmias without relying on alarm prompts.

Participants also described a progression from mechanical textbook matching and reliance on senior colleagues toward a cognitive model that integrates intuitive recognition with systematic evidence validation, with several reporting identifiable breakthrough moments during training in which clinical reasoning became qualitatively transformed. New graduate programs should therefore extend ECG training through progressive, multi-week modules, with each stage focusing on increasingly complex rhythm interpretations using real patient cases from the unit [35]. Given that this cognitive transformation appeared to be facilitated by real-time, contextualized correction rather than deferred feedback, charge nurses should establish peer mentoring partnerships where experienced staff observe newer nurses during actual patient care [36], intervening immediately when suboptimal ECG interpretation occurs rather than waiting for shift debriefings. Staff development departments should maintain databases of actual ECG strips from unit patients, organized by complexity level, enabling targeted practice for nurses at different competency stages, while simulation labs should replicate the specific monitoring equipment used in each unit to ensure direct transfer to daily practice.

#### 4.1.2 Implications for policy-making and healthcare system management.

Participants demonstrated a transition from hesitant, anxiety-driven responses toward confident and proactive clinical engagement, including the capacity to initiate clear diagnostic communication and recommend interdisciplinary action, suggesting that current institutional competency benchmarks may not adequately reflect the response capability that effective cardiac monitoring requires. Hospital administrators should therefore mandate minimum ECG interpretation competency standards for critical care nurses, requiring demonstrated proficiency in recognizing life-threatening arrhythmias within clinically appropriate timeframes, though nursing assessment must carefully integrate this with comprehensive clinical indicators [37]. Human resources policies should incorporate advanced ECG skills into job descriptions and performance evaluations, with salary differentials for nurses achieving specialized cardiac monitoring certifications. Quality improvement departments should track nursing-initiated emergency responses for cardiac events as key performance indicators, measuring the interval from rhythm change to appropriate intervention.

Beyond technical competency, participants described a reconceptualization of the nursing role itself, from task-oriented order execution toward active clinical collaboration, patient advocacy, and intrinsic commitment to ongoing professional development, indicating that institutional structures should be designed to sustain and reinforce this expanded professional identity rather than reduce ECG monitoring to a procedural obligation. Healthcare systems should therefore establish regional RCDP training centers where nurses from multiple hospitals can access standardized, high-fidelity simulation equipment and expert instructors. Insurance and regulatory bodies should recognize hospitals with certified ECG-competent nursing staff through reduced malpractice premiums and accreditation advantages. Management should allocate dedicated time for ongoing ECG skills maintenance, protecting training hours from reassignment to direct patient care during staffing shortages. Budget planning should include regular technology updates for ECG simulation equipment and protected educator positions to ensure consistent training quality across all critical care units.

#### 4.1.3 Implications for future studies.

Future investigations should address several practical research needs. (1) Multi-site comparative studies should examine RCDP implementation across diverse hospital systems and international settings to establish broader implementation guidelines and assess improvements in patient outcomes across varied healthcare environments. (2) Longitudinal investigations should track individual nurses over extended periods following training to identify factors sustaining competency over time and determine optimal refresher training intervals, while examining the relationship between enhanced ECG skills and career advancement patterns. (3) Future mixed-methods designs should triangulate self-reported experiential accounts with objective performance indicators, including pre/post ECG interpretation assessments and simulation-based behavioral observation, to strengthen the evidentiary basis for competency and transformation claims.

## 5 Limitations

This study has the following potential limitations: (1) The single-center design with thirteen participants limits generalizability across diverse healthcare systems and cultural contexts where hierarchical structures, educational frameworks, and professional role expectations may differ from those of the study environment. (2) Potential investigator bias arose from the primary researcher simultaneously implementing RCDP training and conducting interviews, creating possible expectancy effects despite reflexive journaling and role clarification measures. (3) Credibility was partially constrained by returning thematic summaries rather than verbatim transcripts to participants, restricting verification to the interpretive level and representing a partial rather than complete credibility strategy. (4) Participant accounts of competency development and transformative growth were self-reported and not independently verified against objective performance measures, limiting the strength of claims regarding behavioral or skill-based change.

## 6 Conclusion

This phenomenological investigation suggested that rapid cycle deliberate practice training grounded in Tanner’s clinical judgment model is associated with perceived multidimensional shifts within the professional consciousness of critical cardiac care nurses, as reflected in their descriptions of perceptual, cognitive, and behavioral patterns encompassing perceived evolution from fragmented data processing toward holistic pattern recognition, cognitive progression from mechanical rule application toward integrated clinical reasoning, behavioral movement from hesitant reactivity toward confident proactivity, and a described professional development toward enhanced ethical responsibility and autonomous practice. These findings suggest that effective electrocardiographic interpretation competency development requires educational approaches that simultaneously address cognitive, behavioral, and identity dimensions of professional growth rather than isolated technical skill acquisition. The practical implications indicate that healthcare institutions should integrate RCDP methodologies into routine staff development, establish competency-based performance standards, and allocate protected time for simulation-based learning activities to enhance patient safety outcomes through improved nursing clinical judgment capabilities. Future research should examine multi-site implementation effectiveness, conduct longitudinal competency retention studies, and investigate technology-enhanced RCDP approaches to optimize learning efficiency and accessibility across diverse healthcare environments.

## Supporting information

S1 Document Semi-structured interview guide. Interview outline and questions used for qualitative data collection. (DOCX)

S2 Table Qualitative study findings. Summary of themes and results from qualitative analysis.
(DOCX)

## Abbreviations

CCU: Cardiac Critical Care Unit
ECG: Electrocardiogram/Electrocardiographic
RCDP: Rapid Cycle Deliberate Practice
