# Supplementary material for: From noticing to reflection: A qualitative exploration of rapid cycle deliberate practice effects on electrocardiographic monitoring judgment in critical cardiac care nurses
Source: PLoS One. 2026 Jul 6;21(7):e0353168. doi: 10.1371/journal.pone.0353168 (PMC13336174; doi:10.1371/journal.pone.0353168)
Supplement: S1 Document — (DOCX) [file pone.0353168.s001.docx]

**Introduction:**

Thank you for participating in this research study. We are interested in your authentic experiences with Rapid Cycle Deliberate Practice (RCDP) in 'ECG monitoring abnormality identification and emergency response,' and how this training has transformed your approach from rule-dependent to intuitive responses. This interview will take approximately 60 minutes, and with your permission, we will record it for academic research purposes only. All information will be de-identified and kept strictly confidential, and you may decline to answer any question or terminate the interview at any time. There are no standard answers—we value the specific contexts and feelings you experienced. Are you willing to participate and consent to recording?

**I. Pre-Training Judgment Foundation and Dependency Patterns**

1.Before participating in the intensive training, when you heard ECG monitoring alarms, where was your attention primarily focused? How would you describe the way you "saw" ECG tracings at that time—did you check items systematically or have some kind of holistic sense? What was typically your first reaction when encountering complex situations? When multiple alarms sounded simultaneously, how did you decide on the priority order for handling them? In the noisy CCU environment, what types of changes were most easily noticed by you, and what were most easily overlooked?

2.What thinking patterns and steps did you primarily follow when analyzing ECG abnormalities at that time? How long did it typically take from hearing an alarm to making a judgment? Which phases were most prone to errors or hesitation? When standard procedures didn't completely match actual situations, how did you usually handle this? When encountering unfamiliar abnormal situations, what did you rely on to make judgments? In time-critical resuscitation situations, what were you most concerned about missing?

**II. Critical Transformations During Intensive Training**

3.Please recall a memorable "interruption-correction-restart" episode from your training. What were your physical and emotional reactions when interrupted on the spot? How did this immediate feedback differ from previous guidance you had received? When did you start feeling things became "smooth"? What type of correction was most enlightening for you—those about technical operations or about judgment approaches? During periods of intensive training, how did you handle feelings of frustration or anxiety?

4.Was there a particular moment during training when you felt you had an "epiphany" or sudden clarity? What was your internal experience at that moment? What specifically changed—did you notice new details, or did your entire perceptual approach become different? Did this transformation happen suddenly or gradually? How would you describe this process? Could this new feeling be consistently reproduced in subsequent clinical practice?

**III. Clinical Application of New Judgment Patterns**

5.When identifying ECG abnormalities now, what do you "grasp" first? How does this perceptual approach fundamentally differ from your previous step-by-step checking? Can you detect subtle changes that you previously wouldn't have noticed? What kinds of details now capture your attention? When you perceive abnormalities but cannot immediately explain them clearly, how do you handle this uncertainty?

6.When your intuitive judgment doesn't completely align with standard procedures, what internal process do you experience? How do you balance trusting your instincts while ensuring patient safety? What circumstances prompt you to return to rule verification mode? How has your experience of "interacting" with monitoring equipment changed compared to before? When intuitive judgments prove incorrect, how do you process these experiences?

7.Please describe an instance where you successfully handled a clinical situation using newly acquired skills. What was your judgment process at that time? In the multi-task CCU environment, how do you maintain sensitivity to ECG monitoring? How do you allocate attention among different tasks? Does the noisy environment affect your judgment? How do you cope with these distractions? In emergency situations, what changes have occurred in your judgment speed?

**IV. Judgment Communication and Ethical Considerations in Team Collaboration**

8.How do you now communicate your ECG monitoring findings to physicians or colleagues? How does this communication approach differ from before? When your judgment differs from that of more senior medical staff, how do you handle these disagreements? In emergency situations, how do you balance concise expression with accurate information transfer? How do patients' families react to your professional judgments? How does this affect your self-perception?

9.With improved judgment capabilities, what new understanding do you have of your role and responsibilities within the CCU team? In ECG monitoring judgment, how do you balance timely alerts with avoiding excessive intervention? When you detect potential problems but are uncertain about their severity, how do you handle this moral pressure? Under what circumstances would you proactively escalate or insist on your judgment? Has this new judgment capability changed your understanding of nursing ethical responsibilities?

**V. Self-Recognition of Professional Development and Future Expectations**

10.How has this training changed your perception of your professional capabilities? What is your confidence level now when facing ECG abnormalities? How have your feelings changed when taking on more judgment responsibilities? What changes have occurred in your role positioning when collaborating with other professionals? How has this capability enhancement influenced your understanding of nursing professional value?

11.What skill development stage do you feel you are currently at? Which aspects have reached relatively mature judgment levels, and which aspects still primarily rely on standard procedures? What are your expectations for continuing to improve ECG monitoring judgment capabilities? Under what circumstances do you still feel uncertain and need to seek support? For training the next generation of CCU nurses, what do you consider most important?

**Conclusion:**

Thank you for your comprehensive sharing. Your experiences will help us better understand the internal processes of nurse skill development and provide evidence for improving clinical education. If you think of other important experiences later, please feel free to contact us at any time. Thank you again for your time and trust.
