# Supplementary material for: From noticing to reflection: A qualitative exploration of rapid cycle deliberate practice effects on electrocardiographic monitoring judgment in critical cardiac care nurses
Source: PLoS One. 2026 Jul 6;21(7):e0353168. doi: 10.1371/journal.pone.0353168 (PMC13336174; doi:10.1371/journal.pone.0353168)
Supplement: S2 Table — Summary of themes and results from qualitative analysis. (DOCX) [file pone.0353168.s002.docx]

| **Theme** | **Sub-theme** | **Nodes** |
| --- | --- | --- |
| Shifting Perceptual Focus | Fragmented Data Perception | Before, when I heard an alarm, my first reaction was to see which patient's bed it was and then rush over to look at the numbers on the monitor. (Q1,P1) |
|  |  | My attention was definitely focused on the monitor, staring at the flashing numbers and waveforms. (Q1,P2) |
|  |  | Before the training, when an alarm sounded, my attention was mainly on the monitor's numerical values, to see which parameter was abnormal. (Q1,P3) |
|  |  | Before the training, my attention was mainly on the monitor's alarm sounds and lights; whichever one rang or flashed, I would go look at it. (Q1,P12) |
|  | Holistic Pattern Perception | Now, when identifying an ECG abnormality, the first thing I "latch onto" is no longer a single waveform, but the overall "rhythm" and "waveform characteristics" of the entire ECG. (Q5,P1) |
|  |  | Now, when I identify an ECG abnormality, the first thing I "latch onto" is the "overall morphology" and "dynamic changes" of the ECG. (Q5,P2) |
|  |  | I tended to look at the ECG more holistically, checking if the overall rhythm was regular and if there were any major distortions in the waveform. (Q1,P4) |
|  |  | Now, when identifying an ECG abnormality, the first thing I "latch onto" is the "overall trend" and "anomalous signs". (Q5,P13) |
| Shifting Cognitive Models | Mechanical Matching and Authority Reliance | My way of looking at an ECG was very mechanical, just matching it to textbook knowledge. (Q1,P3) |
|  |  | In complex situations, my first reaction was to ask for help from a senior teacher. (Q1,P5) |
|  |  | When encountering an unfamiliar abnormality, what did you rely on to make a judgment? For unfamiliar abnormalities, I primarily relied on the experience of senior nurses. (Q2,P11) |
|  |  | My way of looking at an ECG was basically just "copying the picture"—I'd match what I saw to the diagrams in the book. (Q1,P12) |
|  | Training Breakthrough Moments | When I shifted my attention from individual waves to the overall rhythm...I suddenly noticed a detail I had never seen before...At that moment, I felt the whole ECG "came alive" before my eyes. (Q4,P1) |
|  |  | This instant feedback made me feel like there was nowhere to hide my mistakes, but it also made me more focused. (Q3,P2) |
|  |  | This instant feedback made me feel like a primary school student, but I had to admit that this method really helped me spot problems faster. (Q3,P3) |
|  |  | When I combined the "static" and "dynamic" aspects of the ECG...I suddenly saw a detail I had overlooked before...I felt I wasn't just looking at a picture, but reading a "story". (Q4,P4) |
|  | Integrating Intuition and Evidence | My approach was to perform a comprehensive analysis, combining the patient's medical history and clinical presentation. (Q1,P10) |
|  |  | Before, it was "judgment by experience"; now, it's "data validation". (Q5,P6) |
|  |  | Now, when identifying an ECG abnormality, the first thing I "latch onto" is an "intuitive judgment" backed by "detailed confirmation". (Q5,P8) |
|  |  | I look at ECGs by combining the patient's vital signs, consciousness level, and even subtle changes in facial expression to make a comprehensive judgment. (Q1,P13) |
| Evolving Response Patterns | Hesitant and Reactive Responses | When things got complicated, like several alarms going off at once, honestly, my mind would just go blank. (Q1,P1) |
|  |  | In complex situations, my first reaction was to be a bit stunned, then I'd quickly look through books or search for information. (Q1,P3) |
|  |  | I hadn't been working for long and lacked experience, so before the training, I was a bit scared when I heard an alarm. (Q1,P5) |
|  |  | When multiple alarms went off at once, I would usually be at a loss, not knowing what to do first. (Q1,P12) |
|  | Confident and Proactive Responses | My heart tightened, but I didn't panic like I used to. I immediately rushed to the bedside. (Q7,P2) |
|  |  | Now when I communicate my ECG findings...I'm more direct and clear...I used to just describe the phenomenon; now I can give a preliminary judgment and suggestion. (Q8,P1) |
|  |  | Now, I'll say, "Patient in P4's monitor shows a coved ST-segment elevation...I recommend a doctor assess for acute myocardial infarction immediately and prepare thrombolytic drugs." (Q8,P4) |
|  |  | In emergencies, my judgment speed has significantly increased because I no longer check things item by item but can quickly recognize patterns. (Q7,P1) |
| Redefined Professional Role | New Professional Identity | I used to see myself as just an executor, doing whatever the doctor said. Now, I feel more like a "sentinel" and a "collaborator." (Q9,P1) |
|  |  | When collaborating with doctors, I feel I'm no longer just a "messenger" but can engage in professional discussions with them and even offer my own perspectives. (Q10,P2) |
|  |  | Now, I see myself more as a "diagnostic assistant" and "risk manager," able to identify problems earlier and provide more professional judgments...to doctors. (Q9,P6) |
|  |  | This improvement in my skills has made me realize that nursing is not just physical labor but also a mental challenge. Our professional value is far greater than I imagined. (Q10,P1) |
|  | Evolving Ethical Responsibility | I would insist on my judgment and escalate a report when I believe I am correct and it involves the patient's life and safety. (Q9,P1) |
|  |  | This new ability has changed my understanding of nursing ethics; I believe nurses are not just there to follow orders, but to think proactively and be responsible for the patient's safety. (Q9,P1) |
|  |  | I now believe nurses must not only execute orders but also think proactively and be accountable for patient safety. (Q9,P3) |
|  |  | This enhanced ability has changed my understanding of nursing ethics. I now believe nurses are not just executors of orders but must also think proactively and take responsibility for patient safety. (Q9,P4) |
|  | Continuous Learning and Mastery | I feel like I'm in a rapid growth phase...To further improve my skills, I hope to delve deeper into electrophysiology to understand the pathological mechanisms behind the waveforms. (Q11,P1) |
|  |  | I feel I'm in a stage of steady improvement...I hope to learn more about the differential diagnosis of ECGs to improve my ability to judge difficult cases. (Q11,P3) |
|  |  | As a CCU specialist nurse, I feel I'm in a "striving for excellence" phase...I hope to participate in more advanced training and academic exchanges to continuously improve my professional level. (Q11,P6) |
|  |  | For training the next generation of CCU nurses, I believe the most important thing is to cultivate their critical thinking and lifelong learning abilities so they can continually adapt to clinical changes. (Q11,P4) |
